# Visualization of Scleral Flap Patency in Glaucoma Filtering Blebs Using OCT

**DOI:** 10.1016/j.xops.2024.100604

**Published:** 2024-08-23

**Authors:** Jeremy C.K. Tan, Matthew Roney, Anshoo Choudhary, Mark Batterbury, Neeru A. Vallabh

**Affiliations:** 1 St Paul’s Eye Unit, Royal Liverpool University Hospital, Liverpool, UK; 2 Faculty of Medicine and Health, University of New South Wales, Kensington, New South Wales, Australia; 3 Department of Eye and Vision Sciences, Institute of Life Course and Medical Sciences, University of Liverpool, Liverpool, UK

**Keywords:** Anterior-segment optical coherence tomography, Aqueous outflow resistance, Deep sclerectomy, Scleral flap, Trabeculectomy

## Abstract

**Purpose:**

To investigate the use of anterior-segment OCT (AS-OCT) to visualize the aqueous outflow pathway and patency of the scleral flap in glaucoma filtration surgery blebs.

**Design:**

Cross-sectional study.

**Subjects:**

Two hundred five filtering blebs of 112 patients with glaucoma who had undergone trabeculectomy (Trab, n = 97) or deep sclerectomy (DS, n = 108) surgery with/without mitomycin-C (MMC).

**Methods:**

Swept-source AS-OCT raster slices were used to image the Trab and DS blebs in sagittal and coronal planes using a standardized protocol. Bleb appearances were classified into 4 categories based on the scleral flap and sclerostomy/trabeculo-descemet window (TDW) appearance: A—sclerostomy/TDW not visible; B—sclerostomy/TDW visible but scleral flap indiscriminate from sclera; C—scleral flap distinct but edges adherent to surrounding sclera; D—scleral flap edges non adherent to surrounding sclera.

**Main Outcome Measures:**

Surgical outcomes were classified into complete success (CS) (intraocular pressure [IOP] ≤18 mmHg with no medications), qualified success (QS) (IOP ≤18 with medications), and failure (F) (IOP >18 mmHg).

**Results:**

The proportions of CS, QS, and F in the Trab and DS cohorts were 45.0% and 29.6%, 33.0% and 31.5%, 22.0% and 38.9% respectively, with a median postoperative follow-up of 8.4 years (standard deviation 7.9; interquartile range 3.2–9.0). In QS and failed blebs, category C (Trab, 53.7%; DS, 52.5%) accounted for the majority of scleral flap appearances, followed by categories A and B. Category D (86.0%; 71.9%) accounted for the majority of appearances in Trab and DS blebs with CS. There was a significantly greater proportion of MMC use in categories C and D compared with categories A and B in both Trab (*P* < 0.0001) and DS (*P* = 0.02) cohorts, demonstrating the association of intraoperative MMC use with increased patency of the scleral flap.

**Conclusions:**

Swept-source AS-OCT may be used to visualize the position and patency of the sclerostomy/TDW and scleral flap in relation to surrounding structures in both sagittal and coronal planes. Although free scleral flap edges are primarily correlated with MMC use, it may also correlate with surgical success. Anterior-segment OCT may be used to complement subjective bleb grading at the slit lamp in the assessment of filtering blebs.

**Financial Disclosure(s):**

The author(s) have no proprietary or commercial interest in any materials discussed in this article.

Reduction of intraocular pressure (IOP) is currently the only known modifiable risk factor for the treatment of glaucoma.^1^ Trabeculectomy (Trab) remains the gold standard in glaucoma filtration surgery (GFS), which is performed to achieve greater IOP reduction or when earlier interventions, including topical medications and selective laser trabeculoplasty, fail to halt glaucoma progression. In Trab surgery, a permanent drainage outflow channel is created for aqueous egress from the anterior chamber to the subtenon’s space.^2^ Nonpenetrating procedures such as the deep sclerectomy (DS) have been developed to reduce the incidence of postoperative complications associated with penetrating forms of GFS like Trab. Deep sclerectomy involves intraoperative dissection down to the trabeculo-descemet window (TDW) followed by removal of a block of deep sclera.^3^ The TDW is the juxtacanalicular meshwork and inner wall of Schlemm canal across which aqueous egresses. Postoperative fibrosis and scarring of the subtenon’s space causes restriction of aqueous flow and over time leads to failure (F). This is a significant clinical challenge in GFS.

The postoperative evaluation of GFS filtering blebs has traditionally relied on clinical grading systems performed at the slit lamp such as the Indiana Bleb Appearance Grading Scale and the Moorfields Bleb Grading System, which document factors associated with surgical success such as bleb area, height and vascularity.^4^ These grading systems have marked variation in the degree of interobserver agreement and are influenced by the experience of the observer.^5^ In blebs with poorly controlled IOP, the anatomical location of resistance to aqueous outflow is often judged by examining bleb morphology prior to intervention.^6^ This partially helps to guide the choice of subsequent intervention which may include needling or same-site revision of the Trab or DS bleb with injection of 5-fluorouracil or mitomycin-C (MMC), a new Trab/DS procedure, aqueous shunt implantation, or cyclodiode laser.^7^^,^^8^ The aim of this study was to explore the use of swept-source anterior-segment OCT (AS-OCT) to visualize the anatomical appearance of the sclerostomy/TDW and scleral flap of filtering blebs in a cross-sectional cohort of patients who had previously undergone Trab and DS surgery. Structural assessment of these components of the aqueous outflow pathway may be potentially used to complement subjective bleb grading in helping clinicians determine how to best manage failed surgical blebs.

## Methods

This was a cross-sectional cohort study conducted at the St Paul’s Eye Unit, Royal Liverpool University Hospital, a tertiary referral eye unit in Liverpool, United Kingdom. The study had the approval of the clinical governance department of the Royal Liverpool University Hospital Trust and adhered to the tenets of the Declaration of Helsinki. Consecutive patients attending the glaucoma clinics of the St Paul’s Eye Unit between December 2022 and June 2023 were consented and recruited. The date of clinic attendance within this period is hereafter referred to as the index visit. The inclusion criteria were adult patients with a diagnosis of primary open angle glaucoma, pseudoexfoliative glaucoma, pigment dispersion syndrome glaucoma, or primary angle closure glaucoma, who had undergone Trab or DS surgery with or without intraoperative MMC ≥12 months before the index visit. Only trabeculectomies or DSs performed as a primary or secondary-site procedure (following a previous filtration procedure) were included; revision of Trab or DS procedures were excluded.

The following clinical parameters at the index visit were recorded: best-corrected logarithm of the minimum angle of resolution visual acuity, IOP on Goldmann applanation tonometry, and number of IOP-lowering medications used in the eye that had filtration surgery. The following information about the prior surgery was recorded from the patient’s historical medical record: date of surgery and use of intraoperative MMC.

### Trab and DS surgical Technique

The Trab and DS surgeries were performed by consultants and clinical fellows under the supervision of consultants who were affiliated with the St Paul’s Eye Unit. The main steps performed in all Trab surgery included the creation of a superior fornix-based conjunctival incision and peritomy, creation of a half-thickness rectangular scleral flap, punch sclerostomy, peripheral iridectomy, suturing of the scleral flap with releasable sutures, and then conjunctival closure. Deep sclerectomy surgery involves creation of a superior fornix-based conjunctival incision and peritomy, a superficial scleral flap, deep scleral flap until exposure of the TDW, excision of the deep scleral flap, and suturing of the superficial scleral flap with interrupted sutures before conjunctival closure. If used intraoperatively, MMC was applied to the sclera following the conjunctival peritomy in both types of surgery.

### AS-OCT Imaging of Filtering Bleb

**Figure 1:**
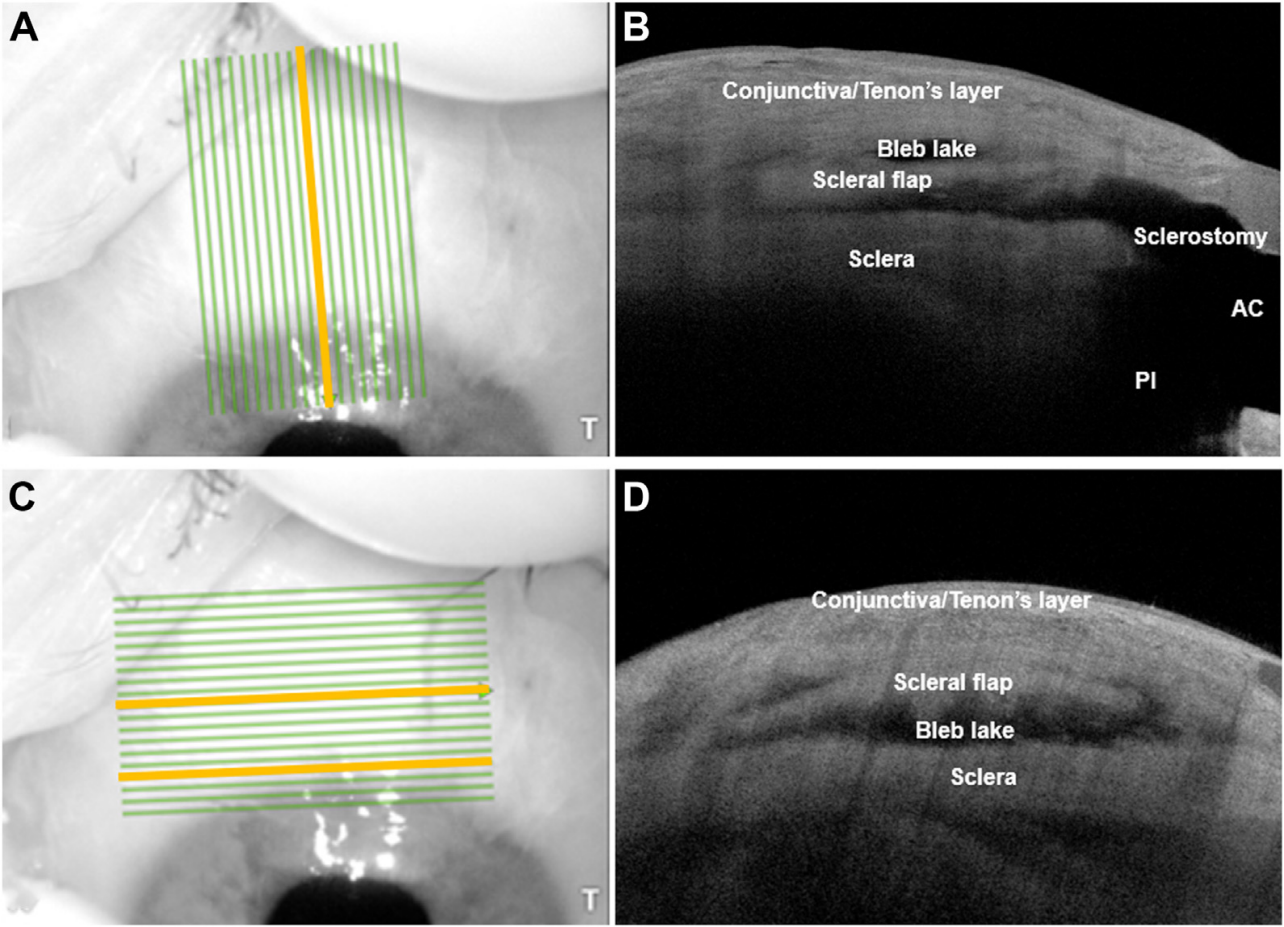
Representative en face and corresponding sagittal (**A** and **B**) and en face and corresponding coronal (**C** and **D**) AS-OCT images of a well-functioning trabeculectomy bleb, with structures labeled. The yellow lines in the en face images (**A** and **C**) represent the slices of interest within the raster block, which captures the anatomy of the scleral flap and sclerostomy within the filtering bleb. AC = anterior chamber; AS-OCT = anterior-segment OCT; PI = peripheral iridotomy.

We have previously published our methodology of imaging DS surgical sites using AS-OCT.^9^ The Trab or DS surgical site (filtering bleb) was first examined at the slit lamp by one of the authors (J.T.). Anterior-segment OCT of the filtering bleb was then performed by the same author using the Anterion (Heidelberg Engineering GmbH) swept-source OCT device. The Anterion uses a laser light source with a wavelength of 1300 nm and at 50 000 Hz to obtain B-scans with axial resolution of 10 microns and transverse resolution of 45 microns. Each scan was performed using the imaging module of the Anterion device using a standardized raster scan measuring 7.5 mm in width and 12 mm in length and comprising 19 slices. The raster block was oriented parallel to the long axis of the scleral flap in the sagittal plane in relation to the bleb (Fig 1B). The anterior limit of the image window was positioned just anterior to the limbus at the peripheral superior cornea, which allows the sclerostomy/TDW, iridocorneal angle, and entire length of the scleral flap to be visualized and captured within the raster slices (Fig 1A, C). The sagittal slice overlying the sclerostomy/TDW was identified from the 19 raster slices (yellow line in Fig 1B).

The raster block was then oriented perpendicular to the initial sagittal plane to visualize bleb structures in the coronal plane. The anterior limit of the raster block was placed anterior to the sclerostomy to allow the full width of the sclerostomy and scleral flap to be visualized and captured within the raster (Fig 1E, F). The coronal slices overlying the sclerostomy and midpoint of the scleral flap were identified from the 19 raster slices (yellow lines in Fig 1D) and exported for analysis. These slices were chosen because they provide a sagittal and coronal cross-sectional view of the sclerostomy/TDW and scleral flap within the filtering bleb.

### Image Processing and Quantification of Surgical Parameters

The filtering bleb images were exported to Matlab (The Mathworks Inc) for image preprocessing in a previously described method.^9^ The following functions were performed on each bleb image sequentially in a standardized manner to improve visualization of the scleral flap: conversion to grayscale, contrast enhancement, thresholding, active contouring, and morphological opening. The maximum bleb height for each Trab and DS bleb was calculated from the sagittal AS-OCT slices.

### Definitions of Surgical Success and Anatomical Level of Aqueous Resistance

The surgical outcome of each Trab and DS case at the index visit was classified into complete success (CS) (IOP ≤18 mmHg with no medications), qualified success (QS) (IOP ≤18 with medications), and F (IOP >18 mmHg or subsequent filtration surgery procedure performed), as per the World Glaucoma Association consensus on definitions of success 2018.^10^

The relative pixel intensities of the filtering bleb structures were enhanced by image processing, which allowed anatomical structures within the bleb to be distinguished from one another. The structural appearance of the scleral flap and sclerostomy/TDW based on the sagittal AS-OCT slice was classified into 4 categories:

A: Sclerostomy/TDW not visible.

B: Sclerostomy/TDW visible, but scleral flap indiscriminate from surrounding sclera.

C: Sclerostomy/TDW visible, scleral flap appearance is distinct, but scleral flap edges appear to be adherent to surrounding sclera.

D: Sclerostomy/TDW visible, scleral flap appearance is distinct, and scleral flap edges appear to be free and nonadherent to surrounding sclera.

**Figure 2:**
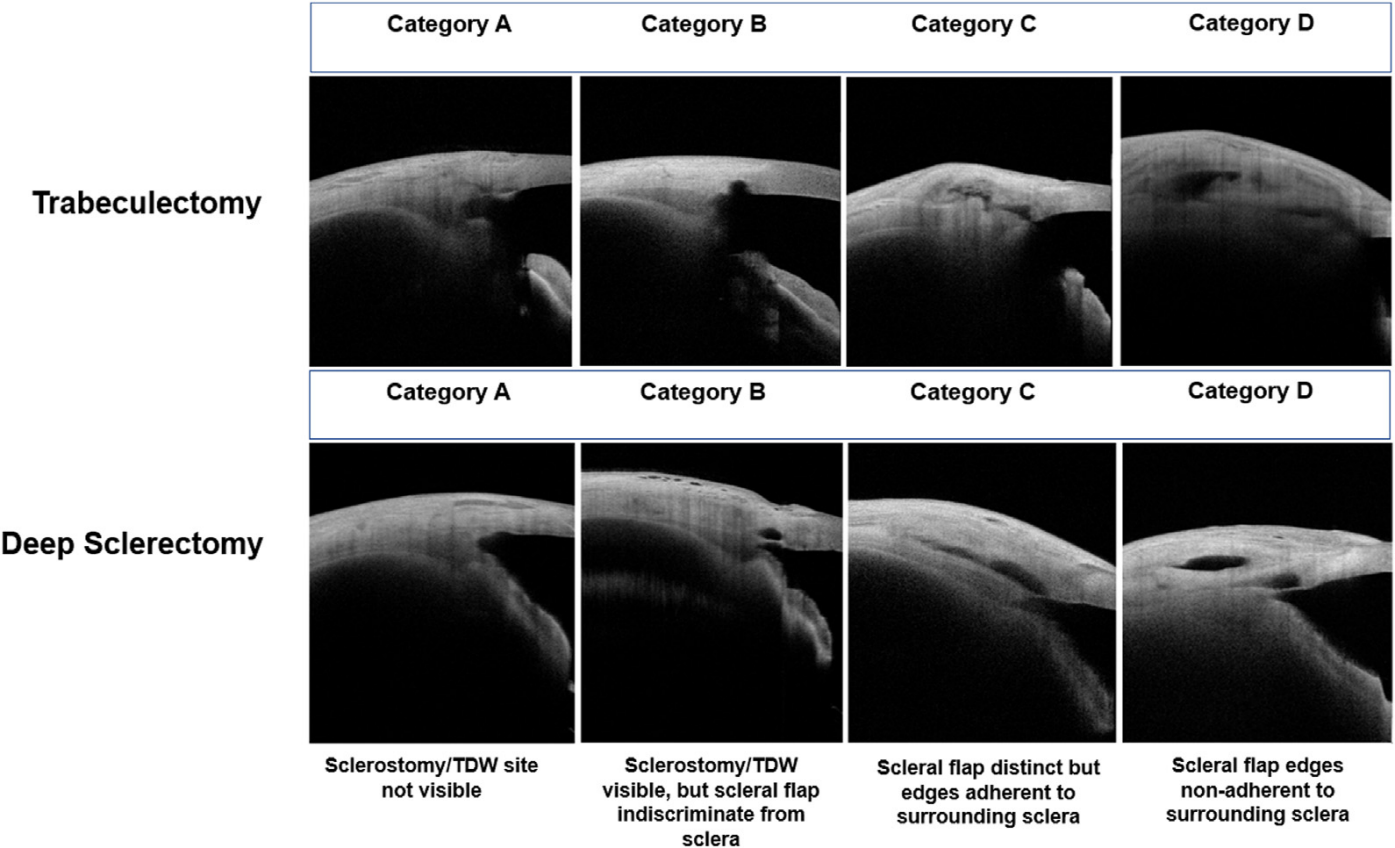
Representative sagittal AS-OCT images of trabeculectomy and deep sclerectomy blebs across the 4 categories of classification of sclerostomy and scleral flap patency, with descriptions of each category in the bottom row. AS-OCT = anterior-segment OCT; TDW = trabeculo-descemet window.

Figure 2 displays representative examples of blebs in the Trab and DS cohorts within each category based on sagittal AS-OCT images. The coronal slices (2 for each bleb) were then used to repeat the above classification, to evaluate the concordance between information gained from sagittal and coronal planes. The classification was performed by 2 authors (J.T. and M.R.) masked to the IOP outcomes, patient records, and the results of the other authors’ findings, with the levels of agreement later compared.

### Statistical Analysis

Descriptive statistics were used to analyze the demographic characteristics of the cohorts. The distributions of quantitative data were first assessed for normality using a D’Agostino and Pearson test of normality, with parametric and nonparametric statistics then applied as appropriate. Analyses were conducted using GraphPad Prism version 10 (GraphPad).

## Results

A total of 205 filtering blebs of 112 patients with glaucoma who had undergone Trab (n = 97) or DS surgery (n = 108) were included in the study. The median postoperative follow-up was 8.4 years (standard deviation 7.9, interquartile range 3.2–9.0). The diagnoses, mean visual acuity, and mean deviation are shown in Table 1.

**Table 1:** Primary Diagnosis, Mean Visual Acuity, and Mean Deviation at the Index Clinic Visit of Trab and DS Cohorts (n = 208)

	Trabeculectomy	Deep Sclerectomy
Diagnoses	N (%)	N (%)
Primary open-angle glaucoma	79 (79)	88 (81.5)
Primary angle-closure glaucoma	12 (12)	9 (8.3)
Pigment dispersion glaucoma	0	4 (3.7)
Pseudoexfoliation glaucoma	1 (1)	2 (1.9)
Other/glaucoma not defined	8 (8)	5 (4.6)
Mean visual acuity in logMAR	0.25 (SD 0.29)	0.21 (SD 0.23)
Mean deviation (MD) in dB	−14.2 (SD 8.5)	−11.5 (SD 7.8)

dB = decibels; DS = deep sclerectomy; logMAR = logarithm of the minimum angle of resolution; SD = standard deviation; Trab = trabeculectomy.

### Proportions of Surgical Success and Scleral Flap Appearance

**Figure 3:**
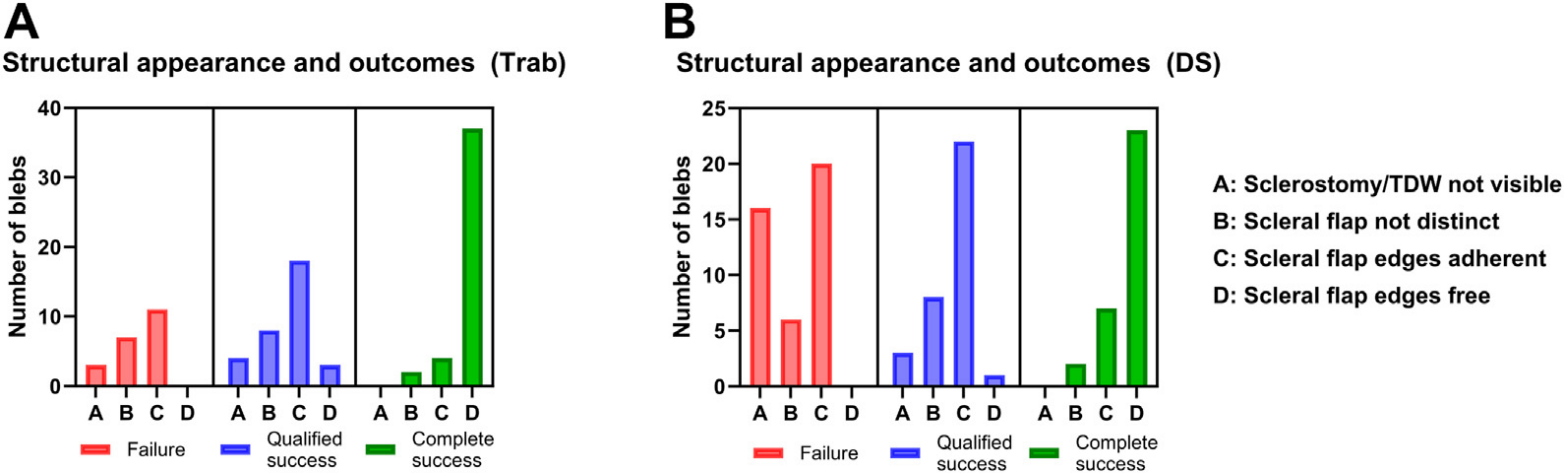
Bar graphs of proportions of blebs within each category of the sagittal AS-OCT classification system of sclerostomy/TDW and scleral flap anatomy in trabeculectomy (**A**) and deep sclerectomy (**B**) cohorts, according to surgical outcome. AS-OCT = anterior-segment OCT; DS = deep sclerectomy; TDW = trabeculo-descemet window; Trab = trabeculectomy.

The proportions of CS, QS, and F in the Trab and DS cohorts were 45.0% vs. 29.6%, 33.0% vs. 31.5%, and 22.0% vs. 38.9% respectively. Table 2 and Figure 3 display the number of blebs at each structural classification category defined on sagittal AS-OCT imaging in failed, QS, and CS blebs. In QS and failed Trab blebs, category C (scleral flap distinct but edges adherent to surrounding sclera; 53.7%) accounted for the majority of scleral flap appearances, followed by category B (sclerostomy visible but scleral flap indiscriminate from sclera; 27.8%) and category A (sclerostomy not distinct/visible; 13.0%). In DS cases, category C accounted for the majority of bleb appearances in QS and failed blebs (52.5%), followed by category A (23.8%) and B (17.5%). Category D (scleral flap edges appear free from surrounding sclera) accounted for the vast majority of appearances in trab (86.0%) and DS (71.9%) blebs with CS. The proportions of categories within each surgical outcome group were significantly different in both Trab (*x*^*2*^ [6, *N* = 97] = 65.32; *P* < 0.0001) and DS (*x*^*2*^ [6, *N* = 108] = 77.75; *P* < 0.0001) cohorts.

**Table 2:** Proportions of AS-OCT Sagittal Classification Category (A–D) Across Surgical Outcomes in Trab and DS Cohorts

	A	B	C	D
Trab (n = 97)				
Failure	3 (3.1)	7 (7.2)	11 (11.3)	0
Qualified success	4 (4.1)	8 (8.2)	18 (18.6)	3 (3.1)
Complete success	0	2 (2.1)	4 (4.1)	37 (38.1)
Total	7 (7.2)	17 (17.5)	33 (34.0)	40 (41.2)
DS (n = 108)				
Failure	16 (14.8)	6 (5.6)	20 (18.5)	0
Qualified success	3 (2.8)	8 (7.4)	22 (20.4)	1 (0.9)
Complete success	0	2 (1.9)	7 (6.5)	23 (21.3)
Total	19 (17.6)	16 (14.8)	49 (45.4)	24 (22.2)

AS-OCT = anterior-segment OCT; DS = deep sclerectomy; TDW = trabeculo-descemet window; Trab = trabeculectomy. The definition for the categories are as follows: (A) sclerostomy/TDW is not visible, (B) sclerostomy/TDW is visible, but scleral flap is indiscriminate from surrounding sclera, (C) sclerostomy/TDW is visible, and scleral flap appearance is distinct, but scleral flap edges appear to be adherent to surrounding sclera, (D) sclerostomy/TDW is visible, scleral flap appearance is distinct, and scleral flap edges appear to be free and nonadherent to surrounding sclera.

### Intraoperative MMC Use and Scleral Flap Appearance

**Figure 4:**
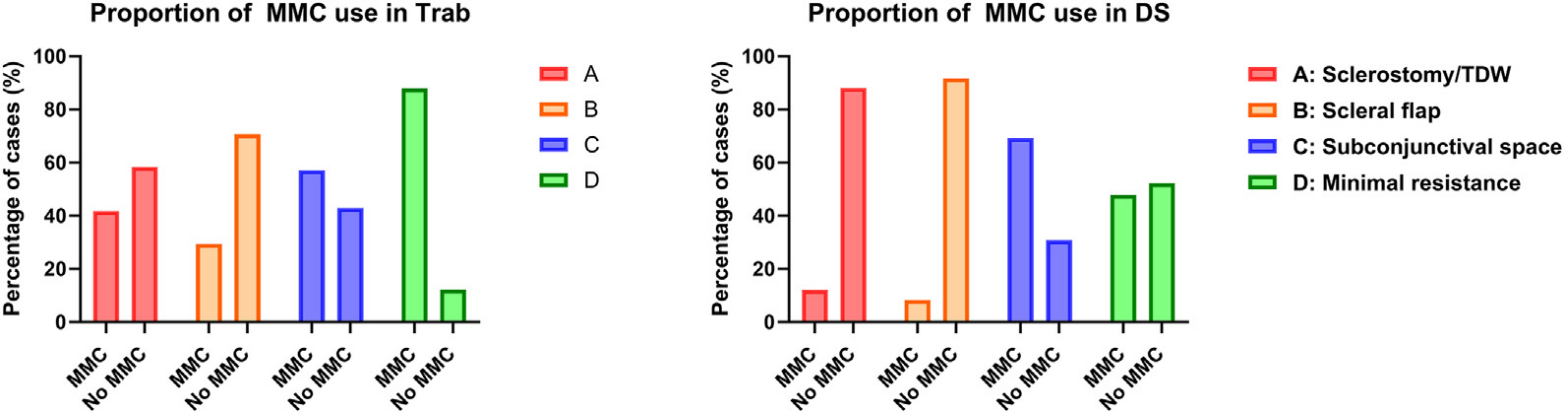
Bar graphs of proportions of intraoperative MMC use within each category of the sagittal AS-OCT classification system of sclerostomy/TDW and scleral flap anatomy in trabeculectomy and deep sclerectomy cohorts. AS-OCT = anterior-segment OCT; DS = deep sclerectomy; MMC = mitomycin-C; TDW = trabeculo-descemet window; Trab = trabeculectomy.

In the Trab group, 56.7% of cases were performed with intraoperative scleral application of MMC, whereas 26.2% of DS cases were performed with MMC. Intraoperative MMC use was significantly greater in the CS group compared with QS and F groups in both the Trab and DS cohorts (Trab: 84.6% vs. 51.6% vs. 33.3% in CS vs. QS vs. F, chi-square, *P* = 0.0003; DS: 40.6% vs. 29.4% vs. 12.2% in CS vs. QS vs. F, chi-square, *P* = 0.02). Figure 4 shows the proportion of MMC use in each structural classification category. There was a significantly greater proportion of MMC use in categories C and D compared with categories A and B in both Trab (*P* < 0.0001) and DS (*P* = 0.02) cohorts, demonstrating the association of intraoperative MMC use with increased patency of the scleral flap.

### Maximum Bleb Height on AS-OCT

**Figure 5:**
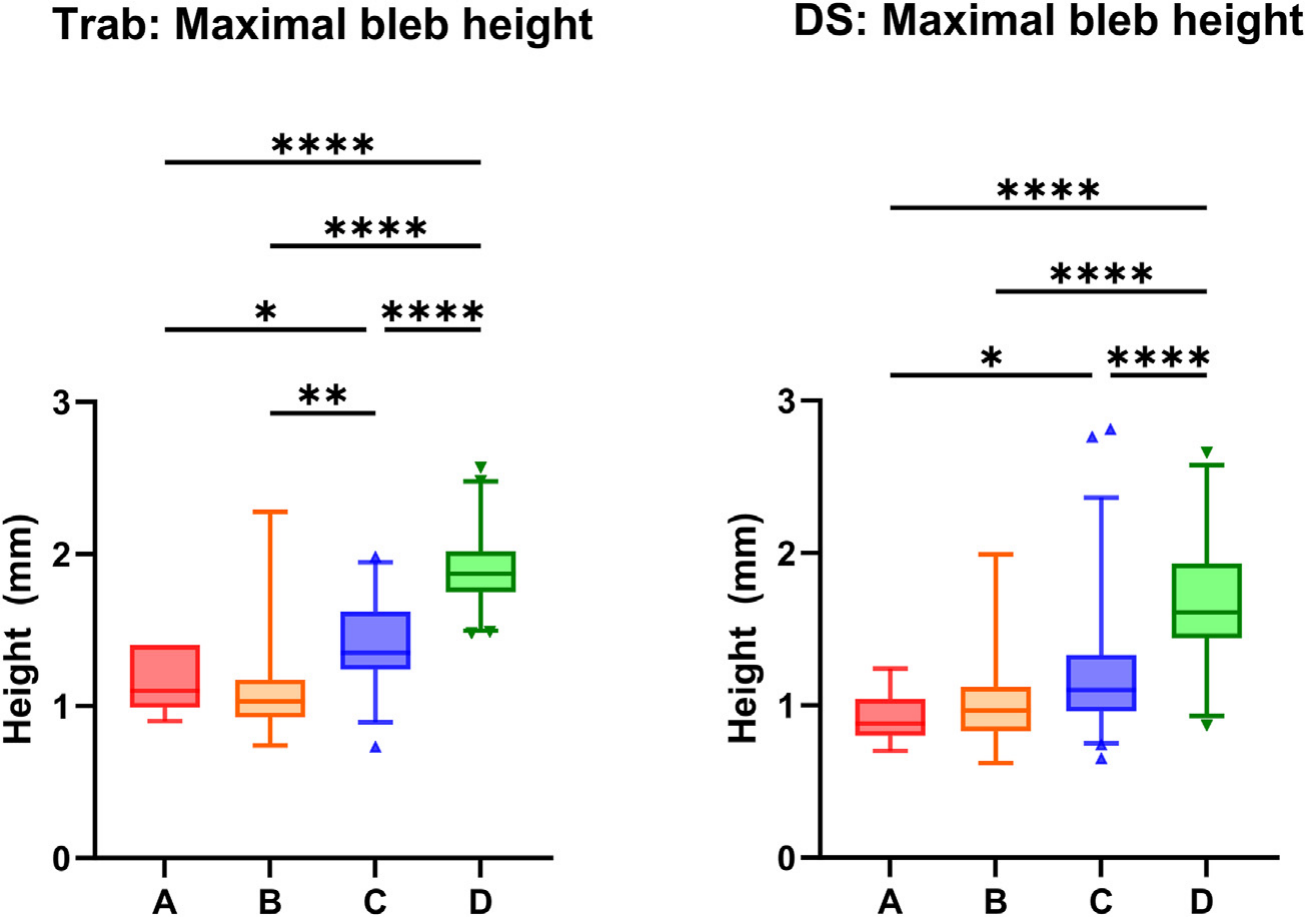
Box and Whisker plots (median, interquartile range, and 5th–95th percentile) and results of 1-way analysis of variance of maximum bleb height across the structural classification categories in the Trab and DS cohorts. Asterisks denote statistical significance (i.e., *P* < 0.05 [∗], < 0.01 [∗∗], < 0.0001 [∗∗∗∗]). Non-significant pairwise comparisons are not shown. Visualizing scleral flap patency following glaucoma filtration surgery. DS = deep sclerectomy; Trab = trabeculectomy.

The maximum bleb height was significantly greater in Trab blebs in category D compared with the other 3 categories. Maximum bleb height in category C blebs was also significantly greater than categories A and B, with no difference in height between the latter 2 categories (1.13 vs. 1.12 vs. 1.42 vs. 1.91 mm in A vs. B vs. C vs. D, 1-way analysis of variance, *F*(3) = 22.8, *P* < 0.0001). In the DS cohort, maximum bleb height was also significantly greater in category D compared with the other 3 categories. There was no difference in height between categories A and B (0.92 vs. 1.04 vs. 1.20 vs. 1.67 mm in A vs. B vs. C vs. D, 1-way analysis of variance, *F*(3) = 15.5; *P* < 0.0001) (Fig 5).

### Interobserver Agreeability and Concordance Between Sagittal and Coronal Classification Systems

The number of observed agreements by the 2 authors who classified the blebs into sagittal classification categories was 154 (75.1%), with a weighted kappa of 0.72 indicating substantial agreement. Blebs with paired sagittal and coronal AS-OCT images were available in 94 trab and 105 DS blebs. The agreement between classification categories was high: Kappa = 0.90 (95% confidence interval 0.83–0.98) in Trab, and Kappa = 0.82 (95% confidence interval 0.73–0.91) in DS (Table 3). Of the blebs with disagreement between sagittal and coronal slices, category B accounted for the majority. In the Trab cohort, 5 blebs with a sagittal appearance of category B had an appearance of category A (3) or category C (2) on coronal slices. In the DS cohort, 8 blebs with sagittal appearance of category B had an appearance of category A (2) or category C (6) on coronal slices. Although the agreement between both sagittal and coronal classification systems were high especially for Trab blebs, the coronal classification system does therefore add more useful information of the status of sclerostomy/TDW and scleral flap.

**Table 3:** Concordance in Classification Categories in Trab and DS Blebs Between the Sagittal (Left Column) and Coronal (Top Rows) AS-OCT Images

Trab		Coronal				Total
		A	B	C	D	
Sagittal	A	4 (4.3)	0	0	0	4
	B	3 (3.2)	13 (13.8)	2 (2.1)	0	18
	C	0	0	31 (33.0)	1 (1.1)	32
	D	0	0	0	40 (42.6)	40
	Total	7	13	33	41	94

DS		Coronal				Total
		A	B	C	D	
Sagittal	A	16 (15.2)	1 (1.0)	0	0	17
	B	2 (1.9)	7 (6.7)	6 (5.7)	0	15
	C	0	4 (3.8)	45 (42.9)	0	49
	D	0	0	0	24 (22.9)	24
	Total	18	12	51	24	105

AS-OCT = anterior-segment OCT; DS = deep sclerectomy; Trab = trabeculectomy.

Numbers and percentage of total in brackets indicate the number of blebs in the category.

## Discussion

A major contributor of F in glaucoma filtration surgery is fibrosis and scarring of the subconjunctival/tenon’s space because of fibroblast proliferation and extracellular matrix deposition, which restricts aqueous flow.^11^ In filtering blebs with poorly controlled IOP, the anatomical location of resistance to aqueous outflow is often judged by examining bleb morphology prior to intervention.^6^ In this study, we demonstrate the use of swept-source AS-OCT to visualize the sclerostomy/TDW and scleral flap anatomy, in a cross-sectional cohort of patients who had undergone Trab and DS surgery. Visualizing the anatomy of the surgically created aqueous outflow pathway could help us better understand the pathophysiology and mechanisms of F.

### Use of AS-OCT in Postoperative Bleb Evaluation

The postoperative evaluation of GFS filtering blebs has traditionally relied on clinical grading systems performed at the slit lamp such as the Indiana Bleb Appearance Grading Scale and the Moorfields Bleb Grading System, which document factors associated with surgical success such as bleb area, height, and vascularity.^4^ The external appearances of bleb morphology used in these systems, however, may not reflect underlying tissue responses. With the advent of improved imaging technology, studies have evaluated the internal microstructure of filtering blebs using techniques such as AS-OCT and ultrasound biomicroscopy.^4^ These modalities can provide quantitative data on bleb microstructure such as bleb wall thickness, presence of microcysts, and measurements of the internal ostium, bleb cavity, and subflap space.^4^^,^^12^, ^13^, ^14^ For instance, Lenzhofer et al^15^ examined 78 eyes of 60 patients post-XEN gel stent implantation, and found that the prevalence of small diffuse cysts was directly associated with lower IOPs, whereas cystic encapsulation at 3 months predicted higher surgical F. Konstantopoulos et al^16^ examined 50 eyes of 50 patients following Trab, DS, or no surgery and found that a tall intrascleral lake and a thick conjunctival/tenon’s layer were associated with good postoperative outcomes as defined by IOP and medication use. In our study, we used the precise localization of OCT raster slices in both sagittal and coronal planes to visualize anatomical details of the drainage outflow channel. Swept-source AS-OCT technology enabled a more precise visualization of these structures than older OCT modalities because of greater penetration from longer wavelengths used, and higher scan speeds.^17^

We found that adherence of the scleral flap to the surrounding sclera accounted for the majority of the structural appearances of the scleral flap in our cohort of Trab and DS blebs that were either failed or had QS. Of note, blebs with greater patency of the scleral flap (categories C and D) had a significantly increased proportion of intraoperative MMC use, which likely indicates the antiscarring effects of MMC use. We also found a high degree of concordance between the sagittal and coronal classification systems, with the latter providing added information for blebs defined as category B based on sagittal AS-OCT slices alone. Using a combination of both sagittal and coronal images may provide the best visualization of the scleral flap and surrounding tissues, which may inform the level of presumed aqueous resistance based on structural appearance.

### Visualizing the Patency of the Scleral Flap May Help Guide Bleb Needling

The anatomical level of Trab aqueous outflow resistance is difficult to establish based on clinical bleb morphology alone. This is, however, often done in clinical practice to guide the choice of bleb needling procedure, of which 2 main types are often performed: “type-1 needling,” which refers to breaking down areas of fibrosis in the subconjunctival/subtenon space and “type-2 needling,” which additionally involves needling under the scleral flap.^6^ A type-3 needling with passage of the needle into the anterior chamber has also been described.^18^ Bleb morphology has been observed to correlate with needling outcomes, with needling appearing to be most effective for cystic or diffuse blebs and less effective for encapsulated or flat blebs.^19^ Tatham et al compared the outcomes of type-1 and type-2 needling procedures and concluded the location of obstruction did not influence outcome, with both needling types found to be equally successful. The anatomic location of aqueous outflow resistance was, however, presumed and not objectively defined: a type-2 needling procedure was performed in cases in which type-1 appeared ineffective during the procedure.^6^ Bleb needling in DS has also been described, with success rates ranging from 64% to 71% at 1 year and 40% to 58% at 5 years in a cohort of 66 eyes.^8^ Our AS-OCT findings show that the appearance and position of the scleral flap correlates with surgical outcomes; the majority of blebs with medication-free success had scleral flaps that appeared to be free from the surrounding sclera. Conversely, blebs with F or QS often had the appearance of the scleral flap being distinct but with edges adherent to the surrounding sclera. Of note, using bleb height alone does not sufficiently help distinguish between underlying bleb morphology in categories A to C.

Visualizing and confirming the patency and position of the scleral flap and sclerostomy may help guide what type of bleb needling is necessary to re-establish aqueous outflow. For instance blebs with a category B appearance may be expected to require needling under the scleral flap given the lack of a visible subflap aqueous lake. Blebs with category A appearance may be more amenable to a more invasive procedure than needling to re-establish flow since even the sclerostomy is visibly patent, such as a bleb revision surgery. A prospective study evaluating the use of this AS-OCT classification system in failed blebs would, however, be needed to assess if the appearances of scleral flap patency correlates with the subsequent outcomes of needling/revision. For instance, AS-OCT should be performed before bleb needling above or under the scleral flap based on the AS-OCT appearance and subsequent outcomes evaluated against the preprocedure and postprocedure OCT bleb morphology on OCT. This may help in validating the utility of AS-OCT for helping guide management of poorly functioning filtering blebs.

### Limitations

Firstly, the cross-sectional nature of the study meant a heterogenous patient population in terms of diagnoses, ethnicity, previous ophthalmic surgery, and postoperative follow-up duration. Although these are known risk factors of F, our primary aim was to demonstrate the use of AS-OCT in a structural assessment of the patency of the sclerostomy/TDW and scleral flap, rather than to evaluate the risk factors of F. Secondly, determination of scleral flap patency was performed manually, subjecting this to inaccuracy and bias. We addressed this by keeping a bank of saved images and having 2 authors perform the classification masked to the IOP outcomes, patient records, and the results of the other authors’ findings. Thirdly, although our findings demonstrate the association between the degree of scleral flap patency and bleb function, as mentioned earlier, a prospective study evaluating the use of AS-OCT in failed blebs is required to validate the proposed classification system. This will serve to verify if the appearances of scleral flap patency correlates well with subsequent outcomes of needling/revision. This is the topic of a future study being planned. Finally, intraoperative MMC use have been shown to improve success rates in GFS.^20^ Because of the long follow-up period, this study captured a number of surgeries performed in the years before the use of intraoperative MMC became widely established, which provided an opportunity for a comparison of outcomes between cases with and without MMC use. The use of MMC will very likely have an impact on the postoperative healing/scarring, which may affect bleb morphology. This is reflected in our findings of a higher proportion of MMC use in blebs in categories C and D.

Swept-source AS-OCT may be used to visualize the position and patency of the sclerostomy/TDW and scleral flap in relation to surrounding structures in both sagittal and coronal planes. Although free scleral flap edges are primarily correlated with MMC use, it may also correlate with surgical success. Anterior-segment OCT may be used to complement subjective bleb grading at the slit lamp in the assessment of filtering blebs.
